# A Facile Synthesis of Red-Shifted Bis-Quinoline (BisQ) Surrogate Base

**DOI:** 10.3390/molecules29174136

**Published:** 2024-08-31

**Authors:** Huda Nazzal, Manoj Kumar Gupta, Amer Fadila, Eylon Yavin

**Affiliations:** School of Pharmacy, Institute for Drug Research, The Hebrew University of Jerusalem, Hadassah Ein-Kerem, Jerusalem 91120, Israel; huda.nazzal@mail.huji.ac.il (H.N.); manojkumar.gupta@mail.huji.ac.il (M.K.G.); amer.fadila@mail.huji.ac.il (A.F.)

**Keywords:** FIT-PNA, surrogate base, cyanine dye, BisQ

## Abstract

Forced intercalation peptide nucleic acids (FIT-PNAs) are DNA mimics that act as RNA sensors. The sensing event occurs due to sequence-specific RNA hybridization, leading to a substantial increase in fluorescence. The fluorophore in the FIT-PNA is termed a surrogate base. This molecule typically replaces a purine in the PNA sequence. BisQ is a surrogate base that connects two quinolines via a monomethine bond. BisQ-based FIT-PNAs have excellent biophysical features that include high brightness and red-shifted emission (λ_em, max_ = 613 nm). In this report, we detail two chemical approaches that allow for the facile synthesis of the BisQ PNA monomer. In both cases, the key compound used for the synthesis of BisQ-CH_2_COOH is the tBu-ester-modified quinoline synthon (compound **5**). Subsequently, one method uses the Alloc acid-protected PNA backbone, whereas the other uses the tBu ester-protected PNA backbone. In the latter case, the overall yield for BisQ acid (compound **7**) and BisQ PNA monomer syntheses was 61% in six synthetic steps. This is a substantial improvement to the published procedures to date (7% total yield). Lastly, we have prepared an 11-mer FIT-PNA with either BisQ or thiazole orange (TO) and studied their photophysical properties. We find superior photophysical properties for the BisQ FIT-PNA in terms of the brightness and selectivity, highlighting the added value of using this surrogate base for RNA sensing.

## 1. Introduction

Peptide nucleic acids (PNAs), DNA analogs developed in the early 90s by Nielsen and co-workers [1,2], have been used for the past three decades as potent antisense molecules for the down-regulation of RNA in vitro and in vivo [3,4,5,6,7,8,9,10,11,12]. In addition, in the early 2000s, PNAs were designed as RNA-sensing molecules by a concept devised by the Seitz group termed forced intercalation PNA (FIT-PNA) [13]. FIT-PNAs are designed by replacing one of the PNA monomers in a given PNA sequence with a fluorophore that is a mono-methine cyanine dye (also known as a surrogate base). In solution, the fluorescence of FIT-PNA, as a single strand, is quenched due to the free rotation of the monomethine bond [14]. However, once hybridized to its complementary RNA/DNA strand, the FIT-PNA fluoresces at the appropriate wavelength of the cyanine dye. The most used surrogate base is thiazole orange (TO). This fluorophore emits at (λ_em,max_ ≈ 535 nm). Other cyanine dyes used in FIT-PNAs include Benzothiazole Orange (BO), Oxazole Yellow (YO), and Oxazolopyridine (JO) [15,16]. All three fluorophores are blue-shifted in comparison to TO. BisQ, developed by our lab for FIT-PNAs [17] and by the Seitz lab for RNA/DNA-based FIT probes (QB, Quinoline Blue) [18], is a red-shifted surrogate base (λ_em,max_ ≈ 602 nm) that has high brightness and quantum yields [19,20,21]. In this report, we describe two alternative synthetic routes to prepare the BisQ PNA monomer; one leading to a 10-fold improvement in the overall yield. We then prepared a model 11-mer FIT-PNA with either TO or BisQ as the surrogate base and compared their sensing properties in terms of the brightness (BR), mismatch discrimination, and binding affinity to complementary RNA.

## 2. Results and Discussion

### 2.1. Synthesis of PNA Backbone

**Scheme 1:**
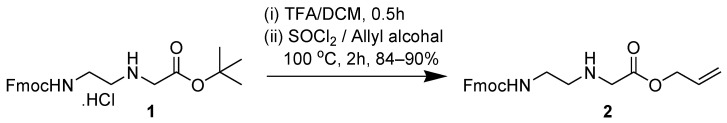
Chemical synthesis of the Allyl–protected PNA backbone as reported by Wickstrom and co-workers [24].

**Scheme 2:**
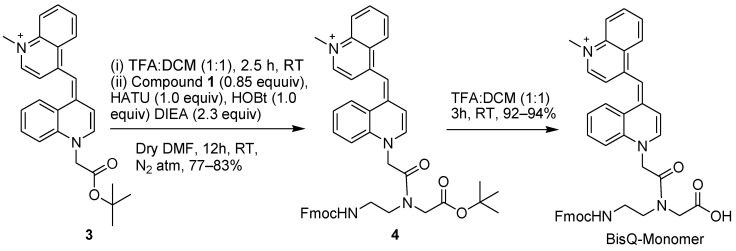
New route for the synthesis of BisQ monomer using the tert–butyl protected PNA backbone (compound **4**, Scheme 1).

The aeg (aminoethyl glycine) PNA backbone is an achiral backbone that may be prepared by several synthetic routes [22,23]. The reported procedure for BisQ is based on eight synthetic steps where the PNA backbone is first coupled to BisQ acid, followed by the removal of both tBOC and tBu ester-protecting groups. The final step is the instillation of the Fmoc group on the primary amine. The overall yield for this synthetic route is ca. 7% [17]. Alternatively, Wickstrom and co-workers [24], reported an alternative route for PNA backbone synthesis (for TO) that is based on the use of the Alloc-protecting group on the C-terminal acid (Scheme 1, Compound **2**). In the newly designed synthesis of BisQ, we have used either the Wickstrom route or a simpler route that is depicted in Scheme 2 that avoids the re-instillation of the Alloc-protecting group. Previously, the motivation for changing the protecting group on the carboxylic acid from tBu ester to Alloc was mainly due to difficulties in the purification of the final compound [24].

### 2.2. Synthesis of BisQ-CH_2_-COOH 

**Scheme 3:**
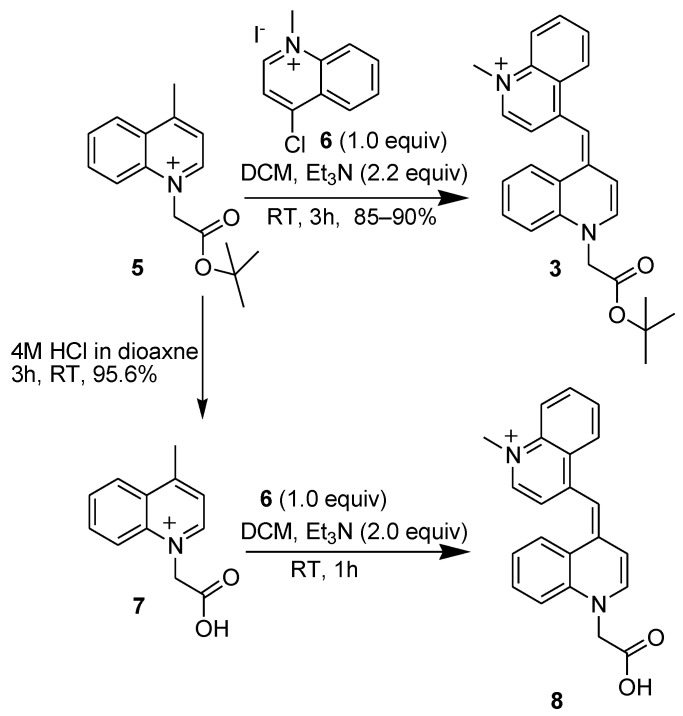
Synthesis of BisQ–COOH (compound **8**) or its tert–butyl ester (compound **3**) via two synthetic routes using the tButyl–protected 4–methyl quinoline (compound **5**).

Previously, BisQ-CH_2_-COOH was synthesized by using the free acid form as bromo acetic acid (BrCH_2_COOH). In our subsequent syntheses of BisQ-CH_2_-COOH, we observed low yields in this step, presumably due to the polymerization of the starting material (BrCH_2_COOH). As an alternative approach, we used the protected acid, namely, BrCH_2_COOtBu. Using this starting material significantly improved the synthesis of the BisQ acid by both methods. BrCH_2_COOtBu was added to 4-methyl quinoline (compound **5**, Scheme 3) and the tBu ester was removed either prior to BisQ acid formation (compound **7**, Scheme 3) or after (compound **8**, Scheme 3). In both methods, high yields were obtained for all synthetic steps.

### 2.3. Synthesis of BisQ PNA Monomer

**Scheme 4:**
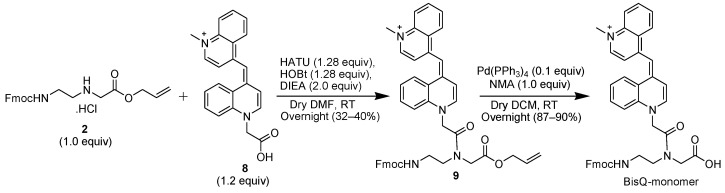
BisQ monomer synthesis via the allyl–protected PNA backbone.

In the first synthetic route (Scheme 4), BisQ acid (compound **8**) was directly coupled to the allyl-protected PNA monomer (compound **2**, Scheme 4). After coupling, compound 9 was purified by normal phase column chromatography, and the purified material was subjected to Pd (Ph_3_)_4_ for the final removal of the Alloc group. The overall yield for this synthetic route (excluding the preparation of the backbone, compound **1**, Scheme 1) was 23%.

In the second synthetic route (Scheme 3), compounds **5** and **6** were added to a DCM solution with triethylamine, affording compound **7** that was purified by normal phase column chromatography. After the tBu ester removal, the TFA salt of BisQ acid was directly coupled to the tBu ester-protected PNA backbone (compound **1**, Scheme 1). Subsequently, compound **4** (Scheme 2) was purified by normal phase column chromatography, followed by the final removal of the tBu ester with TFA. The overall yield for this synthetic route (excluding the preparation of the PNA backbone, compound **1**, Scheme 1) was 61%.

### 2.4. Synthesis and Biophysical Studies on a Model 11-Mer FIT-PNA

We selected an 11-mer FIT-PNA sequence (Table 1) in order to compare the biophysical properties of BisQ with a well-documented cyanine dye, TO. These FIT-PNAs were prepared with a short cationic peptide (dK_4_) as a means to improve their water solubility [25]. Both FIT-PNAs were prepared on the solid support as previously reported [17].

**Figure 1:**
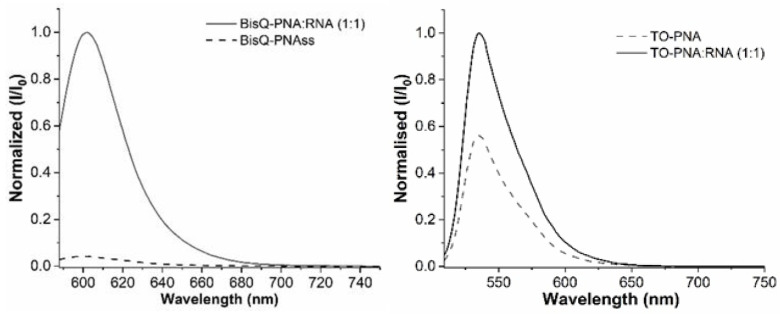
Fluorescence emission spectrum (normalized values) for BisQ-FIT-PNA (**left**) and TO-FIT-PNAs (**right**) towards synthetic RNA. For BisQ-FIT-PNA, λex = 580 nm; λem = 588 nm. For TO-FIT-PNA, λex = 499 nm; λem = 509 nm. [TO-FIT-PNA] = [RNA] = 0.5 μM and [BisQ-FIT-PNA] = [RNA] = 1 μM.

Fluorescence spectra were recorded for both FIT-PNAs before and after annealing to a fully complementary RNA (Figure 1). As opposed to BisQ FIT-PNA, the fluorescence enhancement for TO-FIT-PNA is only ca. 2-fold (I/I_0_ = 1.85, Table 1).

**Figure 2:**
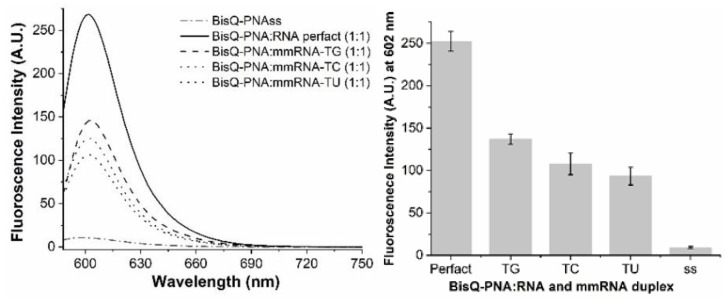
Mismatch discrimination for BisQ-FIT-PNA. FIT-PNA was hybridized to fully complementary RNA (perfect match) or to RNA with a single mismatch (TG, TC, and TU). (**Right**)—Full fluorescence spectrum. (**Left**)—Maximum fluorescence for each duplex (SD based on 3 repeats). [BisQ-FIT-PNA] = [RNA] = 1 μM.

**Figure 3:**
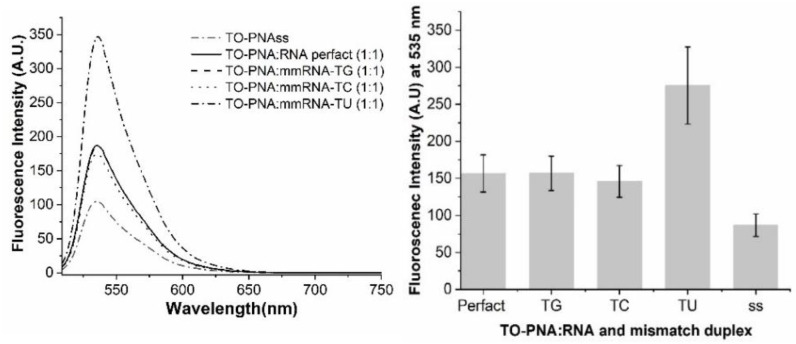
Mismatch discrimination for TO-FIT-PNA. FIT-PNA was hybridized to fully complementary RNA (perfect match) or to RNA with a single mismatch (TG, TC, and TU). (**Right**)—Full fluorescence spectrum. (**Left**)—Maximum fluorescence for each duplex (SD based on 3 repeats). [TO-FIT-PNA] = [RNA] = 0.5 μM.

We next examined the sequence selectivity of both FIT-PNAs by introducing a single mismatch at the flanking base (5′) to the surrogate base (Figure 2 and Figure 3).

BisQ-FIT-PNA shows a robust selectivity for all mismatches tested. However, TO-FIT-PNA has no selectivity to these mismatches. Moreover, for the TU mismatch, the fluorescence of this duplex is higher by almost 2-fold in comparison to the fully matched duplex (Figure 3). One may speculate that the uridine base that is opposite and adjacent to TO does not quench TO fluorescence due to the lack of a primary amine on this base. This may be verified, in the future, by testing other TO-FIT-PNA sequences.

From Table 1, it is clear that BisQ-FIT-PNA is a brighter and more selective RNA probe. The brightness (BR) is ca. 4.3-fold higher for BisQ-FIT-PNA compared to TO-FIT-PNA. This is manifested by a lower extinction coefficient and a lower quantum yield. It is important to note that this comparison was studied for a single 11-mer FIT-PNA sequence and one may not conclude that these dramatic differences will be valid for any given FIT-PNA.

Given the broad use of FIT-PNAs for diagnostics in various diseases, including cancer [17,24,26], RNA editing [20], and infectious diseases [16,21,27], the ease of BisQ synthesis reported in this paper will allow research groups from a variety of fields to readily synthesize and explore BisQ FIT-PNAs and other BisQ-based dyes [28].

## 3. Materials and Methods

### 3.1. Chemicals and Reagents

RNA oligos were purchased from IDT (Coralville, IA, USA). Fmoc/Bhoc-protected PNA monomers were purchased from PolyOrg Inc. (Leominster, MA, USA). Fmoc-protected amino acids and reagents for solid-phase synthesis were purchased from Merck (Darmstadt, Germany), Chem-Impex (Wood Dale, IL, USA), and Bio-Lab LTD (Jerusalem, Israel). Tertbutyl bromoacetate and 4-methylquinoline were purchased from Thermo Fischer (Waltham MA, USA). 4-chloroquinoline was purchased from AABlocks (San Diego, CA, USA).

### 3.2. Chemical Synthesis of BisQ Monomer

*Tert-butyl N-[2-(N-9-fluorenylmethoxycarbonyl) aminoethyl] glycinate hydrochloride* (compound **1**). Compound **1** was synthesized as previously reported [29]. ^1^H NMR (500 MHz, DMSO-*d*_6_) δ 9.36 (s, 2H), 7.89 (d, *J* = 7.5 Hz, 2H), 7.70 (d, *J* = 7.5 Hz, 2H), 7.59 (t, *J* = 5.8 Hz, 1H), 7.42 (td, *J* = 7.4, 1.1 Hz, 2H), 7.33 (td, *J* = 7.4, 1.1 Hz, 2H), 4.33 (d, *J* = 6.8 Hz, 2H), 4.22 (t, *J* = 6.9 Hz, 1H), 3.86 (s, 2H), 3.34 (q, *J* = 6.2 Hz, 2H), 3.00 (t, *J* = 6.4 Hz, 2H), 1.45 (s, 9H); ^13^C NMR (500 MHz, DMSO) δ 165.65, 156.27, 143.80, 140.74, 127.64, 127.08, 125.17, 120.14, 83.01, 65.63, 47.23, 46.65, 46.38, 36.59, 27.62; HRMS *m*/*z* calculated for C_23_H_29_N_2_O_4_ [M + H]^+^ 397.212; 397.211.

Compound **2** was synthesized as previously described [24].

*Bis-quinoline-O^t^Bu ester* (compound **3**). A combination of compound **5** (1.0 g, 3.87 mmol), compound **6** (691 mg, 3.87 mmol), and triethylamine (862 mg, 8.51 mmol) in 60 mL of dry DCM was stirred for 3 h at room temperature, resulting in the formation of a blue solution. After 3 h, 30 mL of water was added, and the aqueous layer was extracted with 3 × 30 mL of DCM. The organic solvent mixture was evaporated, dehydrated using anhydrous Na_2_SO_4_, filtered, and concentrated. The crude product was purified using silica gel column chromatography (Eluent 4–6% MeOH in DCM) to afford a blue compound **3** (1.36 g, 3.41 mmol, 85–90.0%). ^1^H NMR (300 MHz, cdcl_3_) δ 8.26–8.11 (m, 3H), 7.93 (d, *J* = 5.9 Hz, 1H), 7.72 (t, *J* = 7.6 Hz, 1H), 7.67–7.56 (m, 2H), 7.55–7.41 (m, 3H), 7.34 (d, *J* = 7.6 Hz, 1H), 7.20 (dd, *J* = 8.9, 2.9 Hz, 1H), 6.79 (s, 1H), 4.95 (s, 2H), 3.97 (s, 3H), 1.45 (s, 9H); ^13^C NMR (300 MHz, cdcl3) δ 166.1, 150.3, 148.2, 143.8, 142.8, 138.4, 138.1, 133.0, 132.4, 126.8, 126.0, 125.8–125.4 (m), 124.9, 117.1, 115.7, 110.7, 108.5, 97.9, 84.2, 55.5, 43.0, 28.1 ppm; HRMS *m*/*z* calculated for C_26_H_27_N_2_O_2_^+^ 399.206; 399.205.

*Fmoc-Aeg-PNA-BisQ-tert-butyl-ester* (**4**). Compound **3** (500 mg, 1.0 mmol) was dissolved in a mixture of 10 mL of TFA:DCM (1:1) at room temperature, and the reaction mixture was stirred for 2.5 h. A volume of 10 mL of toluene was added, and the solvent was co-evaporated under vacuum. Then, 10 mL of diethyl ether was added, and the solvent mixture was evaporated twice under vacuum. This resulted in the TFA salt of compound **3**, which was used in the next steps without further purification. 

TFA salt of compound **3** was dissolved in 10 mL of dry DMF. Then, HATU (381 mg, 1.0 mmol), HOBt (135 mg, 1.0 mmol), and DIPEA (149 mg, 1.15 mmol) were added. The mixture was stirred at room temperature for 10 min. This reaction mixture was slowly added to a mixture of the HCl salt of Fmoc-aeg-OtBu (compound **1**, 338 mg, 0.85 mmol) and DIPEA (149 mg, 1.15 mmol) in dry DMF (5 mL). The reaction mixture was stirred under an inert atmosphere (argon) for 12 h. Once the reaction was finished (followed by TLC), 10 mL of water was added to the reaction mixture and extracted with 3 × 40 mL DCM. The combined organic layers were then washed with NaHCO_3_ (30 mL) and saturated brine (3 × 30 mL). The organic layers were dried using anhydrous Na_2_SO_4_, filtered, and then concentrated under vacuum. After obtaining the crude product, it was purified using silica gel chromatography (5–6% MeOH in DCM). This resulted in the formation of a blue sticky compound **4** (550 mg, 0.83 mmol, 77–83% yield). ^1^H NMR (500 MHz, DMSO-*d*_6_); δ 8.73 (d, *J* = 8.6 Hz, 1H), 8.65 (d, *J* = 8.6 Hz, 1H), 8.34 (d, *J* = 7.2 Hz, 1H), 8.01–7.94 (m, 1H), 7.89 (dt, *J* = 9.4, 4.7 Hz, 3H), 7.79–7.63 (m, 5H), 7.61–7.46 (m, 2H), 7.44–7.38 (m, 3H), 7.35–7.26 (m, 4H), 5.55 (s, 1H), 5.28 (s, 1H), 4.39 (d, *J* = 6.9 Hz, 1H), 4.35 (d, *J* = 6.5 Hz, 1H), 4.31 (d, *J* = 7.0 Hz, 1H), 4.23 (tt, *J* = 12.8, 7.4 Hz, 2H), 4.13 (s, 2H), 3.97 (s, 1H), 3.74 (s, 1H), 3.57 (s, 1H), 3.23 (d, *J* = 6.2 Hz, 1H), 3.15 (dd, *J* = 14.4, 5.2 Hz, 1H), 2.90 (d, *J* = 7.9 Hz, 1H), 1.45 (s, 6H), 1.38 (s, 3H); ^13^C NMR (500 MHz, DMSO) δ 168.9, 166.7, 166.3, 156.5, 156.3, 156.2, 150.0, 148.2, 144.1, 143.8, 143.3, 143.1, 140.7, 138.6, 132.9, 132.2, 128.9, 127.6, 127.3, 127.1, 126.6, 125.9, 125.6, 125.4, 125.1, 124.6, 121.4, 120.1, 117.9, 117.2, 109.7, 107.6, 96.9, 82.6, 82.4, 81.0, 65.5, 65.3, 54.4, 53.8, 49.9, 48.8, 48.0, 47.3, 46.9, 42.0, 37.3, 27.7 ppm. HRMS *m*/*z* calculated for C_45_H_45_N_4_O_5_^+^ 721.338; 721.337.

*1-[2-(1,1-Dimethylethoxy)-2-oxoethyl]-4-methyl quinolinium* (compound **5**). 4-methylquinoline (715.95 mg, 5 mmol) and tert-butyl bromoacetate (975.3 mg, 5 mmol) in 12.5 mL of acetonitrile were stirred for 24 h at 50 °C. The solvent was evaporated, and the brownish residue was recrystallized in acetone and vacuum dried. The solid was further washed with acetone affording compound **5** as a white solid (1.226 g, 4.75 mmol, 95%). ^1^H NMR (500 MHz, Deuterium Oxide) δ 8.96 (d, *J* = 6.1 Hz, 1H), 8.34 (dd, *J* = 8.5, 1.3 Hz, 1H), 8.13–8.02 (m, 2H), 7.90–7.83 (m, 2H), 5.71 (s, 2H), 2.91 (d, *J* = 0.8 Hz, 3H), 2.12 (s, 1H), 1.35 (s, 9H). ^13^C NMR (500 MHz, Deuterium Oxide) δ 166.09, 161.62, 148.63, 137.59, 135.92, 129.84, 129.11, 127.09, 122.56, 117.95, 86.36, 58.29, 27.02, 19.85. Calculated M.W. for C_16_H_20_NO_2_^+^ 258.14886; M.W. 258.14814.

*1-Methyl-chloroquinolinium iodide* (compound **6**). Compound **6** was synthesized as previously described [17], with a 92% yield.

*1-Carboxymethyl-4-methylquinolinium* (compound **7**). Compound **5** (645.3 mg, 2.5 mmol) and HCl (22 mL, 4M in 1,4-dioxane) were stirred for 3 h at room temperature. The solvent mixture was evaporated under reduced pressure and the resulting solid was washed with diethyl ether and collected by filtration, affording compound **7** (483 mg, 2.39 mmol, 92–96%). ^1^H NMR (500 MHz, Deuterium Oxide) δ 8.92 (d, *J* = 6.1 Hz, 1H), 8.29–8.23 (m, 1H), 8.07–7.97 (m, 2H), 7.83–7.75 (m, 2H), 5.70 (s, 2H), 2.85 (d, *J* = 0.8 Hz, 3H). ^13^C NMR (500 MHz, Deuterium Oxide) δ 169.31, 161.33, 148.51, 137.57, 135.71, 129.71, 129.09, 126.90, 122.49, 118.06, 57.91, 19.73. Calculated M.W. for C_12_H_12_NO_2_^+^ 202.08626; M.W. 202.08521.

*4-[[1-Carboxymethyl-4(1H)-quinolinylidene] methyl]-1methyl quinolinium* (compound **8**). Compound **8** was synthesized as previously described [17].

Compound **9** was synthesized as previously described, by utilizing Compound **2** and **8** [26].

*Final BisQ monomer* (via allyl ester deprotection). This reaction was synthesized as previously described [27].

*Final BisQ monomer* (via tBu ester deprotection). Compound **4** (300 mg, 0.42 mmol) was dissolved in a 12 mL mixture of TFA and DCM in a 1:1 ratio. The solution was stirred for 3 h at room temperature. The solvent was then co-evaporated with toluene (2 × 12 mL) and further with diethyl ether (2 × 12 mL) to obtain a dark blue solid (260 mg, 0.39 mmol, 90–94% yield). ^1^H NMR (500 MHz, DMSO-*d*_6_) δ 8.90 (s, 1H), 8.74 (dd, *J* = 8.6, 4.3 Hz, 1H), 8.66 (d, *J* = 8.6 Hz, 1H), 8.34 (dd, *J* = 7.2, 5.2 Hz, 1H), 8.01–7.94 (m, 1H), 7.90 (dd, *J* = 7.6, 6.2 Hz, 3H), 7.78–7.66 (m, 4H), 7.61–7.53 (m, 2H), 7.51–7.39 (m, 4H), 7.37–7.26 (m, 4H), 5.55 (s, 1H), 5.33 (s, 1H), 4.40–4.35 (m, 2H), 4.31 (d, *J* = 7.0 Hz, 1H), 4.23 (dq, *J* = 14.5, 6.8 Hz, 1H), 4.14 (d, *J* = 2.5 Hz, 2H), 4.03 (s, 1H), 3.90 (s, 1H), 3.59 (d, *J* = 6.7 Hz, 1H), 3.44–3.36 (m, 2H), 3.18–3.12 (m, 1H), 3.02 (td, *J* = 6.7, 4.4 Hz, 1H); ^13^C NMR (500 MHz, DMSO-*d*_6_): major rotamer: δ 171.2, 170.3, 168.2, 166.7, 166.3, 156.4, 149.9, 148.3, 144.1, 143.8, 143.4, 143.1, 140.8, 138.6, 132.9, 129.5, 127.7, 127.1, 126.6, 125.9, 125.6, 125.4, 125.1, 124.7, 120.2, 117.9, 109.7, 109.6, 107.6, 96.9, 65.6, 47.0, 46.7, 42.1, 36.6; minor rotamer: 156.6, 156.2, 150.0, 148.3, 144.1, 143.9, 140.7, 138.7, 132.2, 129.9, 127.9,117.2, 46.7, 46.5, 42.0 ppm. HRMS calculated for C_41_H_37_N_4_O_5_^+^ 665.275; 665.273.

### 3.3. FIT-PNA Synthesis

Manual solid-phase synthesis was performed in 5 mL polyethylene syringe reactors (Phenomenex, Torrance, CA, USA) that were equipped with a fritted disk. HPLC purifications and analysis were performed on a Dionex UltiMate 3000 HPLC system (ThermoFisher Scientific, Waltham, MA, USA) using a semi-preparative C18 reversed-phase column (Jupiter C18, 5 μm, 300 Å, 250 × 10 μm, Phenomenex, Torrance, CA, USA). Eluents A (0.1% TFA in water) and B (Acetonitrile) were used in a linear gradient with a flow rate of 4 mL/min. Mass analysis of FIT-PNAs was performed by Maldi-TOF MS (Microflex, silver edition, Brucker, Billerica, MA, USA).

### 3.4. Tm Analysis

Melting curves (Tm) of the FIT-PNA/RNA duplexes were estimated from UV melting curves measured on an Evolution One Plus UV-Vis Spectrophotometer (Thermo Fisher Scientific, Waltham, MA, USA). FIT-PNAs and their complementary RNAs (1:1.5 ratio) were prepared in PBS buffer (pH 7.0) and adjusted to a final duplex concentration of 2.5 µM. Prior to analysis, the samples were heated from 20 °C to 90 °C at a rate of 5 °C/min and then cooled to the starting temperature at a rate of 2 °C/min. The change in absorbance was monitored at 260 nm by increasing the temperature to 90 °C at a rate of 1 °C/min.

### 3.5. UV-Vis Titrations and Fluorescence Measurements for Determining Quantum Yields

The measurement of the fluorescence quantum yield of FIT PNA:RNA duplexes followed the same protocol as previously reported [30]. The absorbance range of the FIT-PNA duplex determined the choice of reference dye. For example, cresyl violet was used as a reference dye for the BisQ PNA/RNA (1:1) duplex, while fluorescein was employed as a reference dye for the TO PNA/RNA (1:1) duplex.

A serial dilution of FIT-PNA/RNA duplex solutions with an OD smaller than 0.1 at the maximum wavelength was measured to verify that the absorbance maxima remained constant. FIT-PNA duplexes with similar heights of absorbance were used to determine the cut point with respect to the reference dye, and this information was used to gather the fluorescence emission spectra (see Supplementary Materials, Figures S9–S12).

The fluorescence quantum yield was calculated by considering the refractive index (RI) value of the solvents, using the integrated emission value and the OD value at the absorbance maxima of the identical samples. To mitigate the impacts of the inner filter, we employed an optical density (OD) value below 0.1. The quantum yield was calculated using the equation, as detailed in the literature [25].

### 3.6. Fluorescence Measurements

Fluorescence spectra of FIT-PNAs with/out synthetic RNA were measured using a Jasco FP-6500 Spectrofluorometer (Jasco Inc. Co., Ltd, Tokyo, Japan) with a band width set to 2.5 nm and a response rate of 0.2 s. For BisQ FIT-PNA, excitation was set to 580 nm and the emission spectra were recorded from 588 nm to 750 nm. For TO FIT-PNA (excitation at 499 nm), emission was recorded between 509 and 750 nm.

Solutions of FIT-PNA/FIT-PNAs (1 μM) and synthetic RNA (1 μM) were prepared in 1× PBS. The hybridization of FIT-PNA/FIT-PNAs with synthetic RNA was performed by annealing at 65 °C for 2 min followed by incubation at 37 °C for 2–3 h at a ratio of 1:1. The RNA sequence used was 5′-UAUGUAUGUUG. RNAs with mismatches were TU mm: 5′-UAUGUUUGUUG; TC mm: 5′-UAUGUCUGUUG; TG mm: 5′-UAUGUGUGUUG.

## 4. Conclusions

In this report, we detail two improved synthetic routes for the synthesis of the BisQ monomer, a red-shifted surrogate base used in the design of RNA/DNA sensors based on the FIT-PNA approach [13]. In comparison to published protocols, one of these synthetic routes that uses the tBu ester aeg-PNA backbone (compound **7**) results in an overall yield of 61.4% (compared to 7%). In addition, a direct comparison of a model FIT-PNA with either BisQ or TO highlights the superior biophysical properties of BisQ FIT-PNA in terms of the brightness, quantum yield, and sequence selectivity.

## Figures and Tables

**Table 1:** The photophysical properties and binding affinities of these FIT-PNAs are summarized.

FIT-PNA Duplex (1:1)	λ_max_	Ɛ [mM^−1^ cm^−1^]	Ex. (nm)	ɸ_F_ [%]	BR [mM^−1^ cm^−1^]	I/I_0_	*T*_m_ [^o^C]
11-mer BisQ PNA/RNA	587	53.62	580	9.33	5	24	45.2 (±0.2)
11-mer TO PNA/RNA	516	21.73	499	5.41	1.17	1.85	49 (±0.2)

## Data Availability

All data are presented within the submitted manuscript.

## Supplementary Materials

The following supporting information can be downloaded at https://www.mdpi.com/article/10.3390/molecules29174136/s1. Figures S1–S6: HPLC chromatograms and Maldi-TOF MS for FIT-PNAs; Figures S7 and S8: Tm profiles for FIT-PNA/RNA duplexes; Figures S9–S12: UV-Vis spectra for determining ε values for Fit-PNA/RNA duplexes; Figures S13–S18: ^1^H NMR and ^13^C NMR of synthesized compounds.
