# Supplementary material for: A Facile Synthesis of Red-Shifted Bis-Quinoline (BisQ) Surrogate Base
Source: Molecules. 2024 Aug 31;29(17):4136. doi: 10.3390/molecules29174136 (PMC11397033; doi:10.3390/molecules29174136)
Supplement: Supplementary file 1 [file molecules-29-04136-s001.zip › molecules-3162967-supplementary.pdf]

A facile synthesis of red-shifted Bis-Quinoline (BisQ) surrogate base

Huda Nazzal [1, #], Manoj Kumar Gupta [1,#] Amer Fadila[1], and Eylon Yavin [1,*]

[1] Institute for Drug Research, School of Pharmacy, The Hebrew University of Jerusalem, Hadassah
Ein-Kerem, Jerusalem 91120, Israel; huda.nazzal@mail.huji.ac.il (H.N.); amer.fadila@mail.hujilac.il (A.F.); manojkumar.gupta@mail.huji.ac.il (M. K. G.)
[#] Equal contribution
* Correspondence: eylony@ekmd.huji.ac.il (E.Y.)

Tables of contents:

Sequences used for studies	Table S1
HPLC chromatograms of FIT-PNAs	Figures S1-S4
Maldi-TOF MS for FIT-PNAs	Figures S5-S6
Thermal melting profiles for FIT-PNA:RNA duplexes	Figures S7-S8
Determination of extinction coefficient for FIT-PNA:RNA duplexes	Figures S9-S12
^{1}H and ^{13}C NMR spectra for compounds 1, 3, 4, 5, 7, and BisQ monomer	Figures S13-S18

Table S1: Sequences used for studies

ID	RNA/PNA sequences	Cal. M.W.	Obs. M.W.
BisQ-FIT-PNA	3' dK_4 ATACAT BisQ CAAC 5'	3619.78 $[M+H]^+$	3619.92
TO-FIT-PNA	3' dK_4 ATACAT TO CAAC 5'	3647.79 $[M+Na]^+$	3649.58
RNA	5' UAUGUA U GUUG 3'		
RNA-TG	5' UAUGU**G** U GUUG 3'		
RNA-TC	5' UAUGU**C** U GUUG 3'		
RNA-TU	5' UAUGU**U** U GUUG 3'		

dK4 = 4 D-lysines, bold letters – mismatches in RNA sequence

HPLC chromatograms of FIT-PNAs

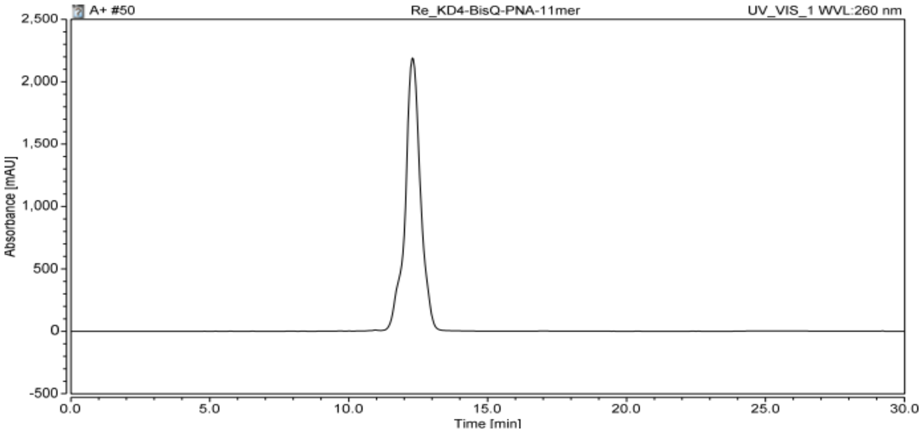

Figure S1: HPLC chromatogram of BisQ FIT-PNA at 260 nm. Eluents: A (0.1% TFA in water) and B (ACN) were used in a linear gradient (5-30% B in 20 min) with a flow rate of 4 mL/min.

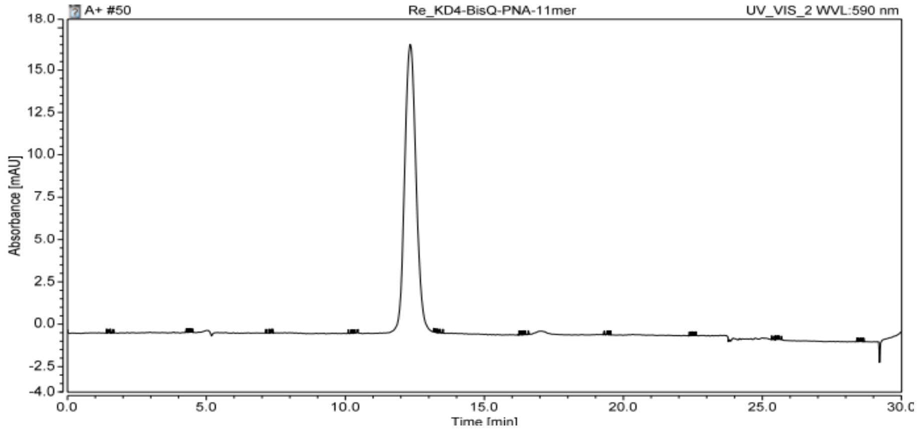

Figure S2: HPLC chromatogram of BisQ FIT-PNA at 590 nm. Eluents: A (0.1% TFA in water) and B (ACN) were used in a linear gradient (5-30% B in 20 min) with a flow rate of 4 mL/min.

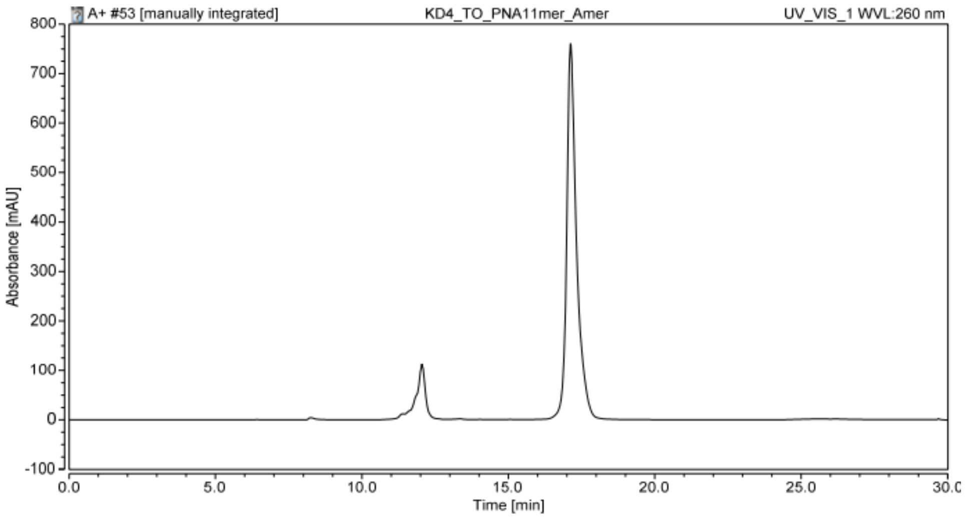

Figure S3: HPLC chromatogram of TO FIT-PNA at 260 nm. Eluents: A (0.1% TFA in water) and B (ACN) were used in a linear gradient (5-30% B in 20 min) with a flow rate of 4 mL/min.

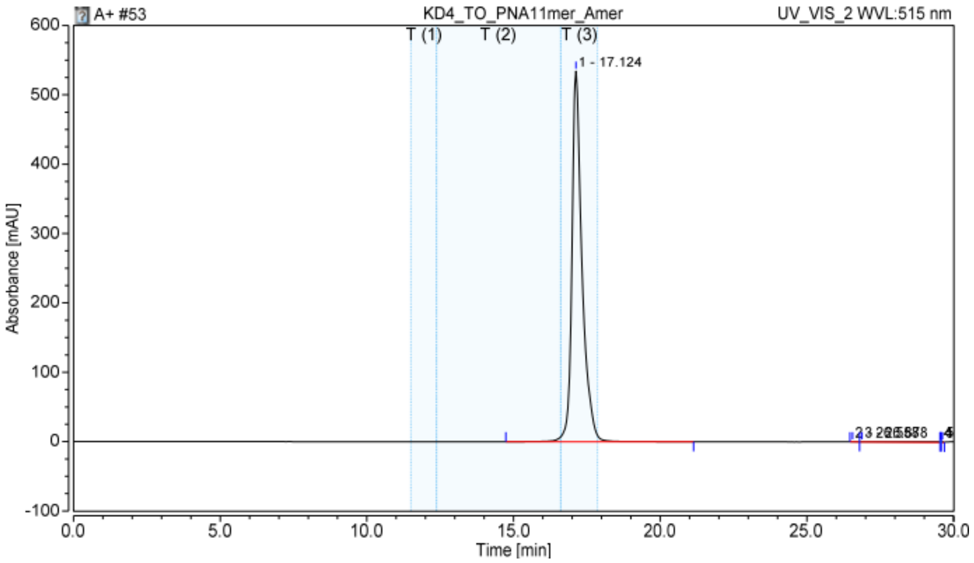

Figure S4: HPLC chromatogram of TO FIT-PNA at 517 nm. Eluents: A (0.1% TFA in water) and B (ACN) were used in a linear gradient (5-30% B in 20 min) with a flow rate of 4 mL/min.

Maldi-TOF MS for FIT-PNAs

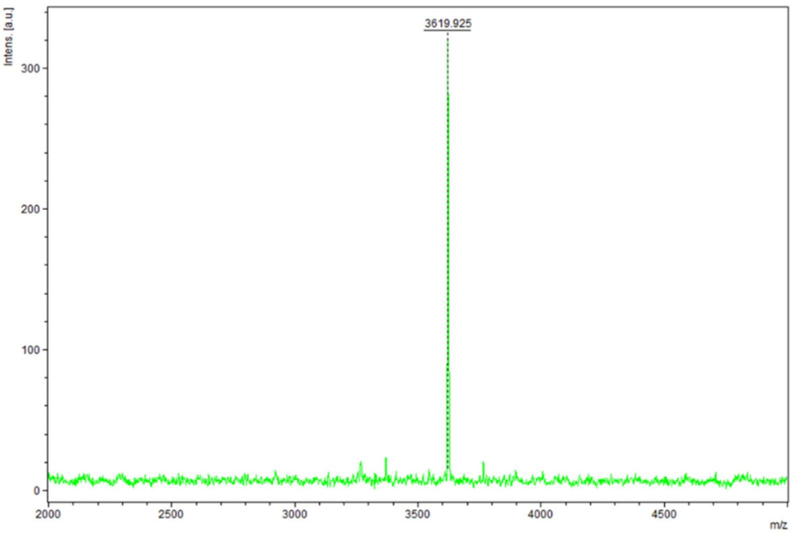

Figure S5: Maldi-TOF for BisQ FIT-PNA. Calculated mass: 3619.78 $[M+H]^+$. Observed Mass: 3619.92.

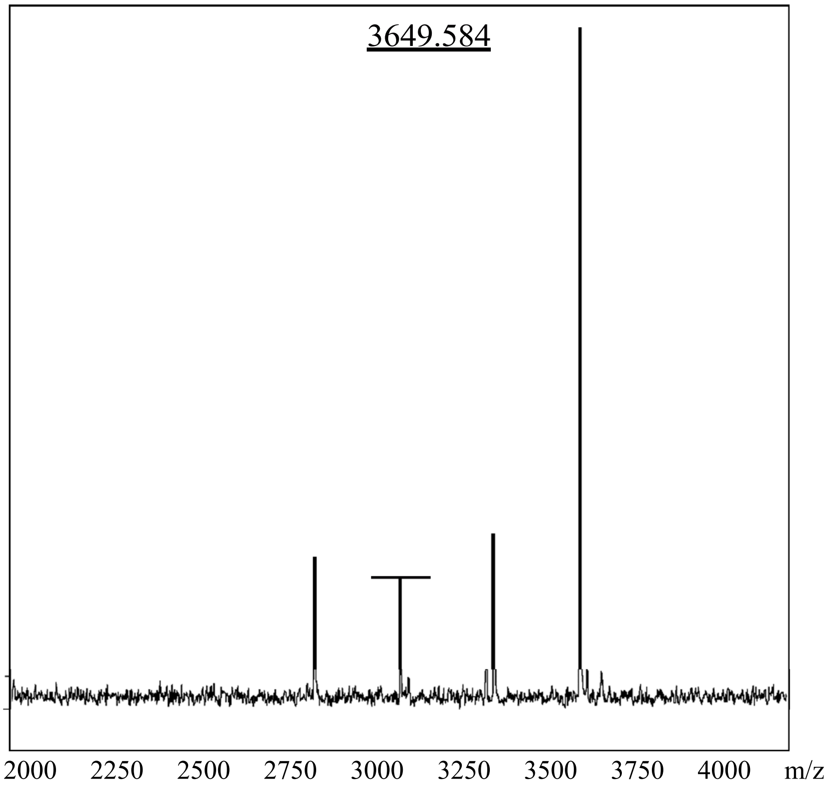

Figure S6: Maldi-TOF for TO FIT-PNA. Calculated mass: 3647.79$[M+Na]^+$. Observed Mass: 3649.58.

Thermal melting profiles for BisQ FIT-PNAs

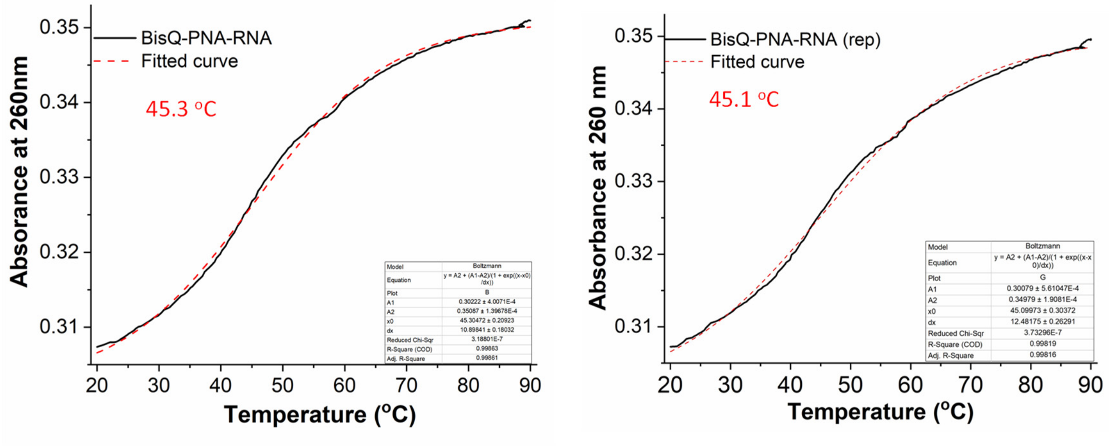

Figure S7: Melting curve profiles for BisQ FIT-PNA annealed to complementary RNA. [BisQ FIT-PNA] = 1 µM, [RNA] = 1.5 µM.

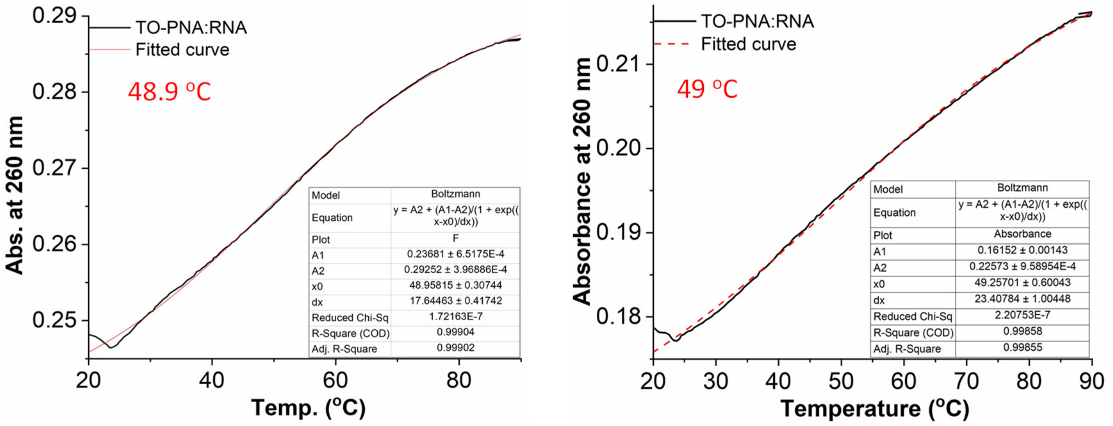

Figure S8: Melting curve profiles for TO FIT-PNA annealed to complementary RNA. [TO FIT-PNA] = [RNA] = 2 µM.

Determination of extinction coefficient for BisQ-FIT-PNA:RNA duplex

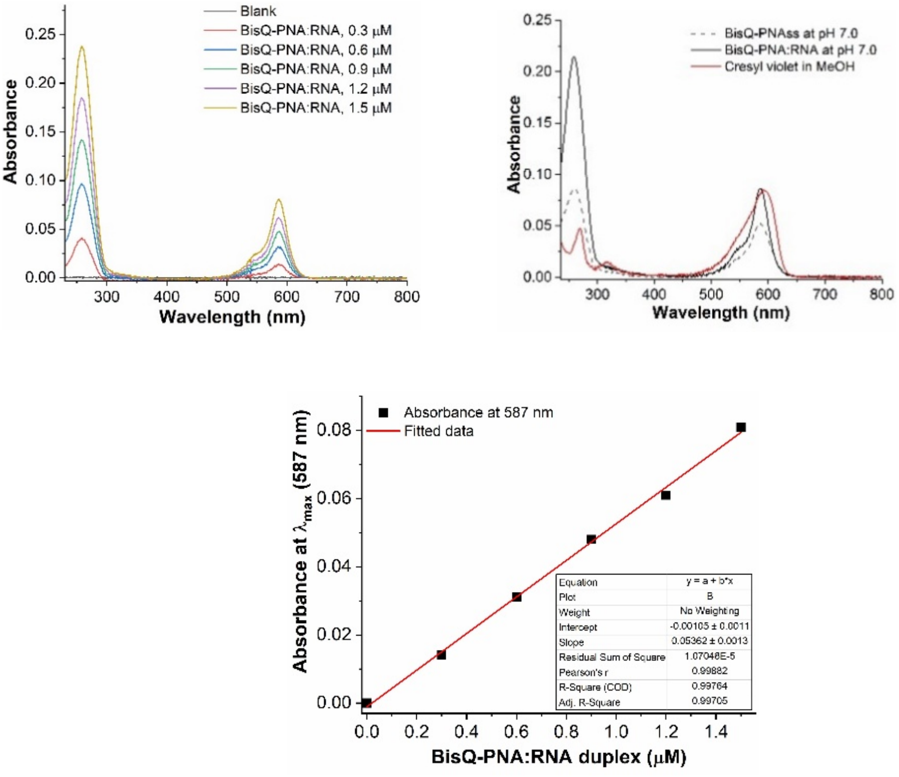

Figure S9: UV titration of BisQ-FIT-PNA:RNA (1:1) duplex for determination of extinction coefficient at absorbance maxima.

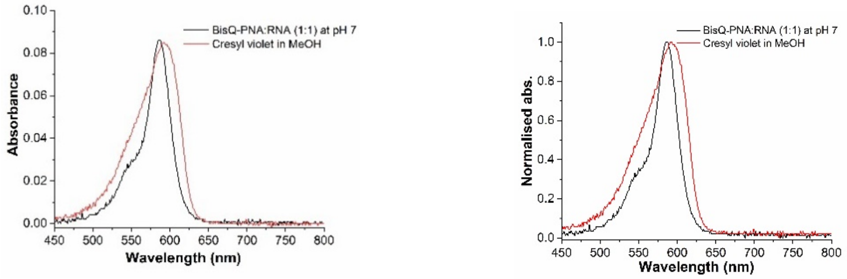

Figure S10: Determination of cut point (excitation wavelength) for BisQ-FIT-PNA:RNA duplex.

Determination of extinction coefficient for TO-FIT-PNA:RNA duplex

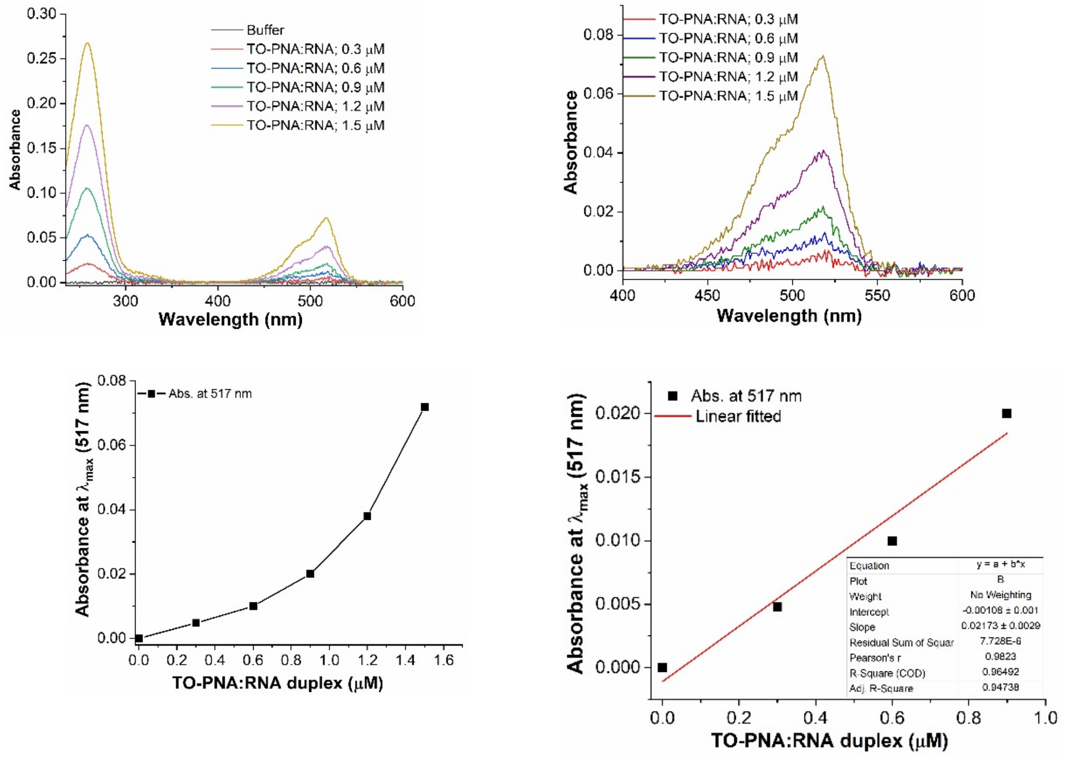

Figure S11: UV titration of TO-FIT-PNA:RNA duplex for determination of extinction coefficient at absorbance maxima. The fitting was done at lower concentrations (0-1 μM) to avoid in filter effect.

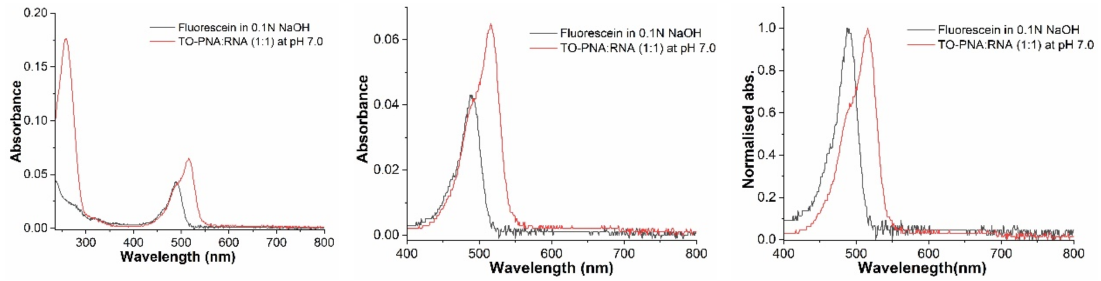

Figure S12: Determination of cut point (excitation wavelength) for TO-FIT-PNA:RNA duplex.

^{1}H and ^{13}C NMR spectra of compound 1

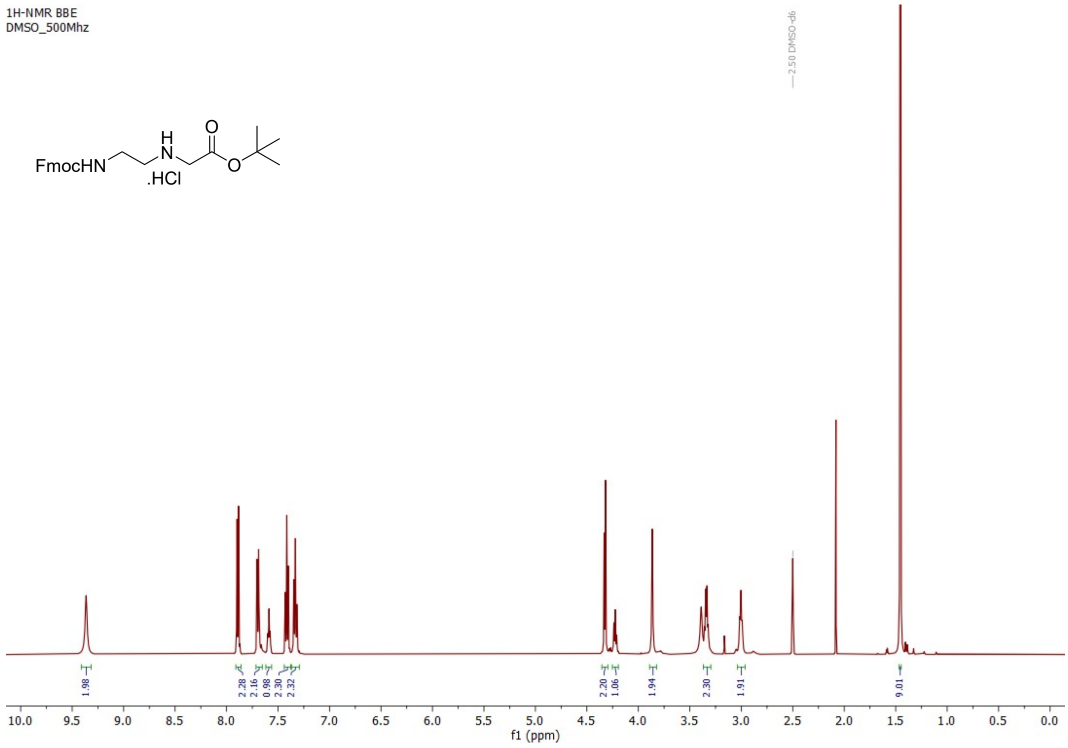

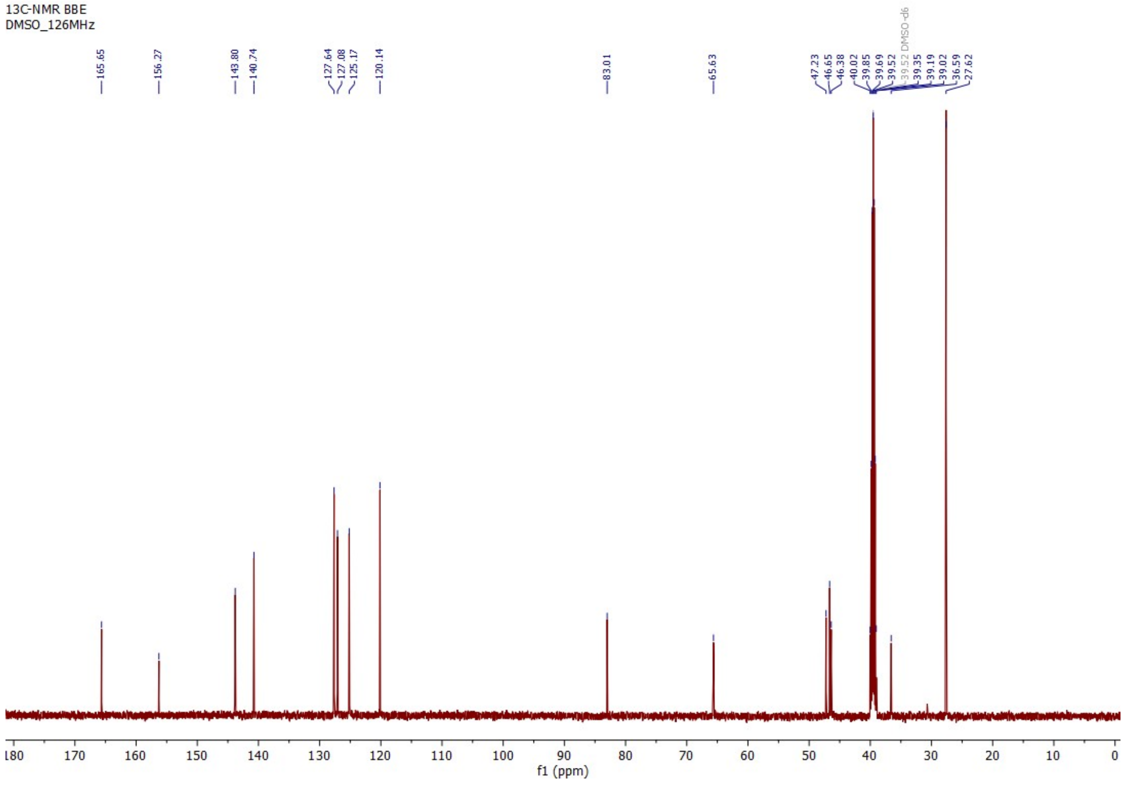

Figure S13: ^{1}H and ^{13}C NMR spectrum of compound **1**

^{1}H and ^{13}C-NMR spectra of compound 3

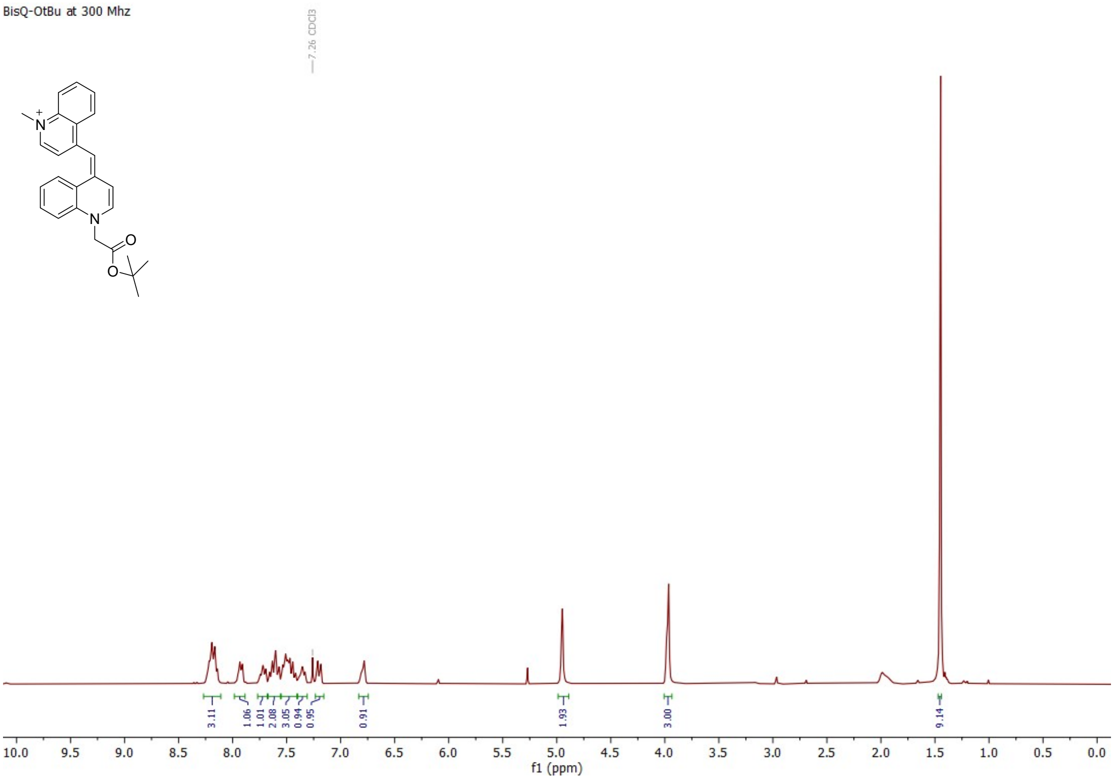

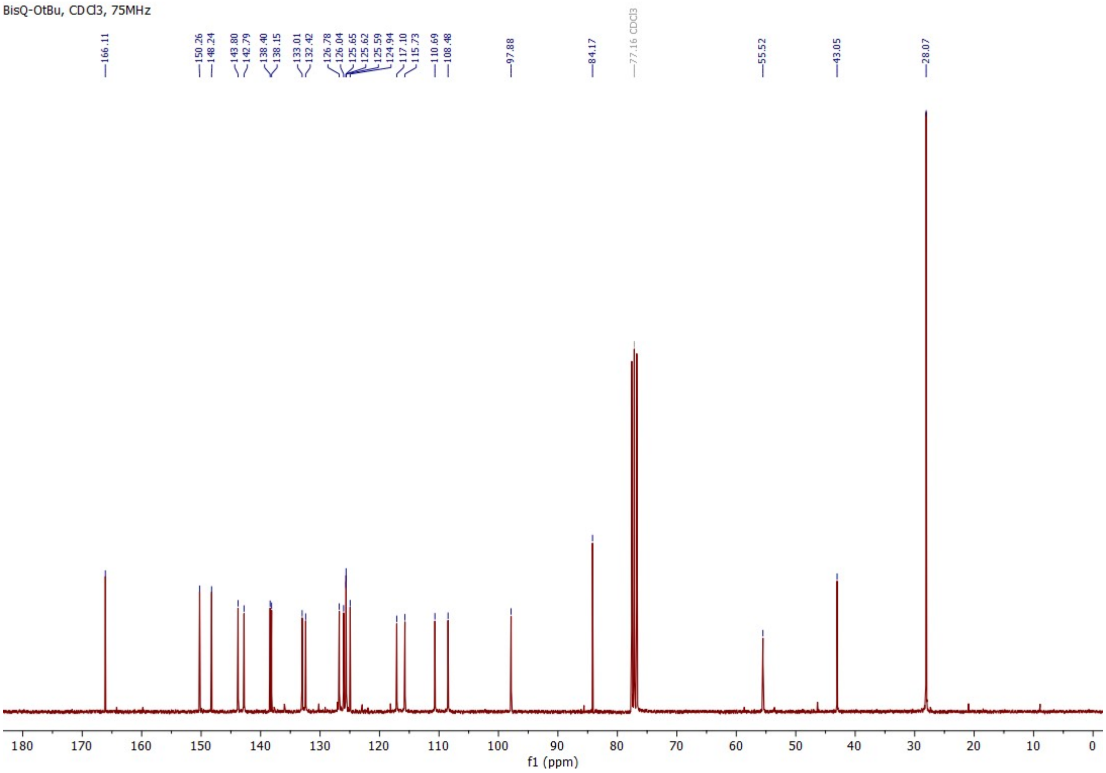

Figure S14: ^{1}H and ^{13}C NMR spectrum of compound **3**

^{1}H and ^{13}C NMR spectra of compound 4

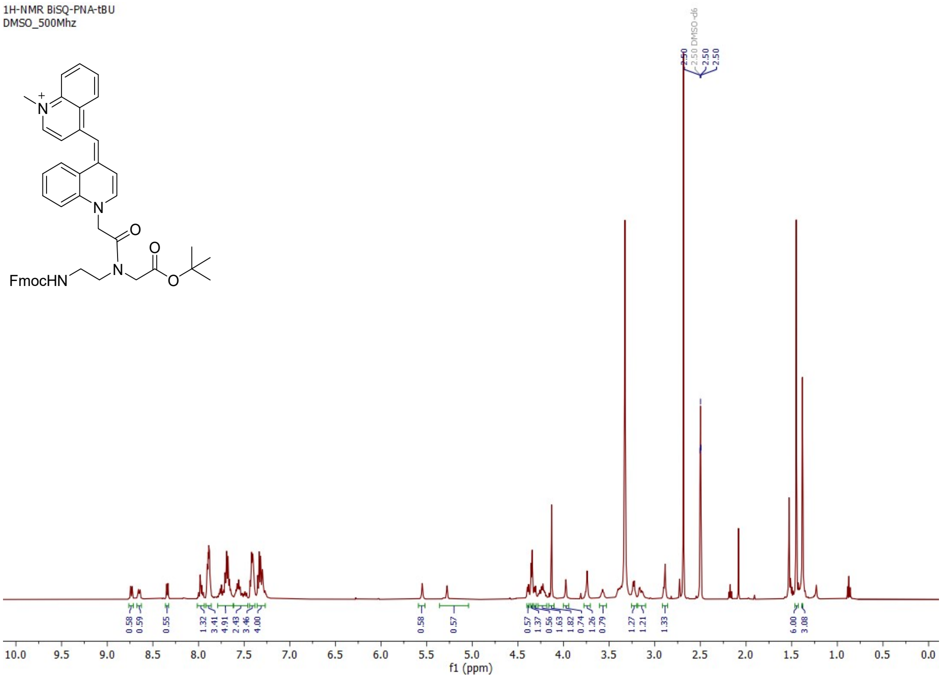

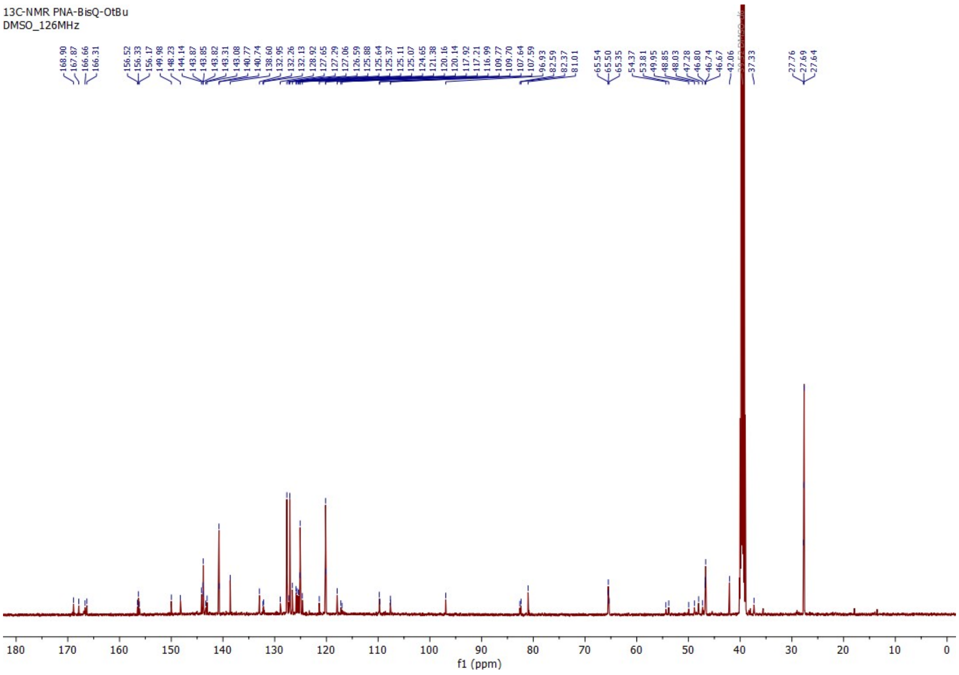

Figure S15: ^{1}H and ^{13}C NMR spectrum of compound **4**

^{1}H and ^{13}C-NMR spectra of compound 5

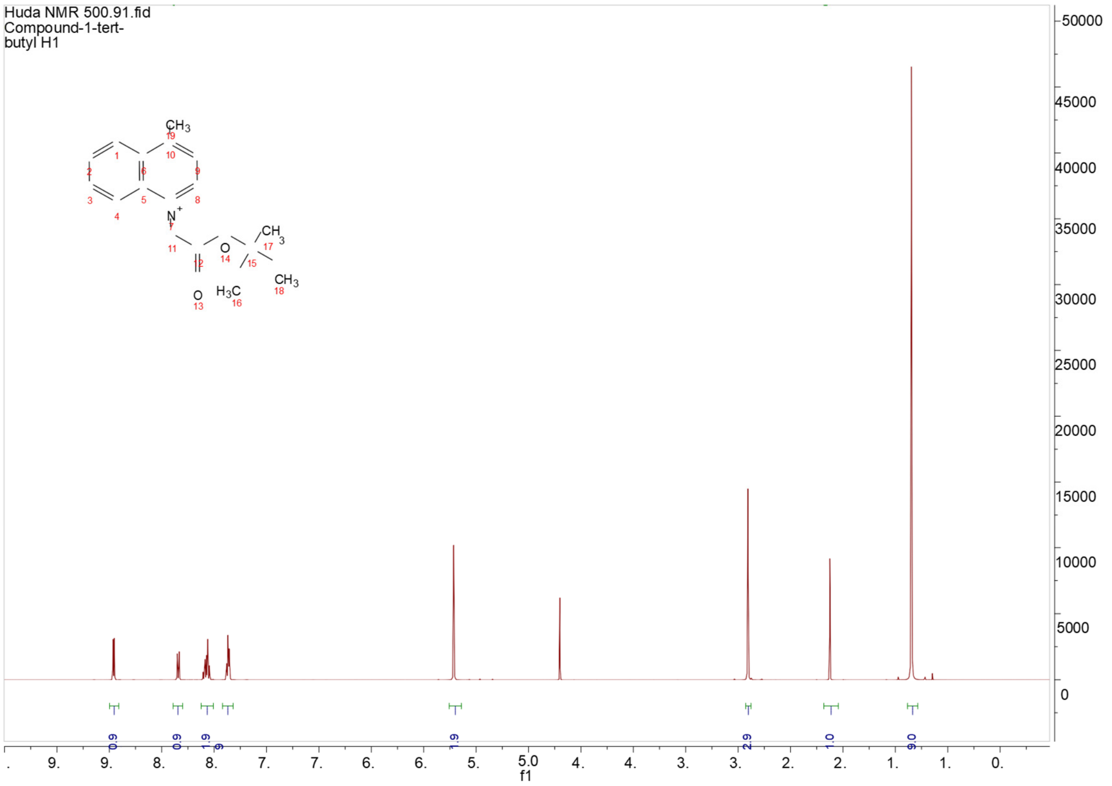

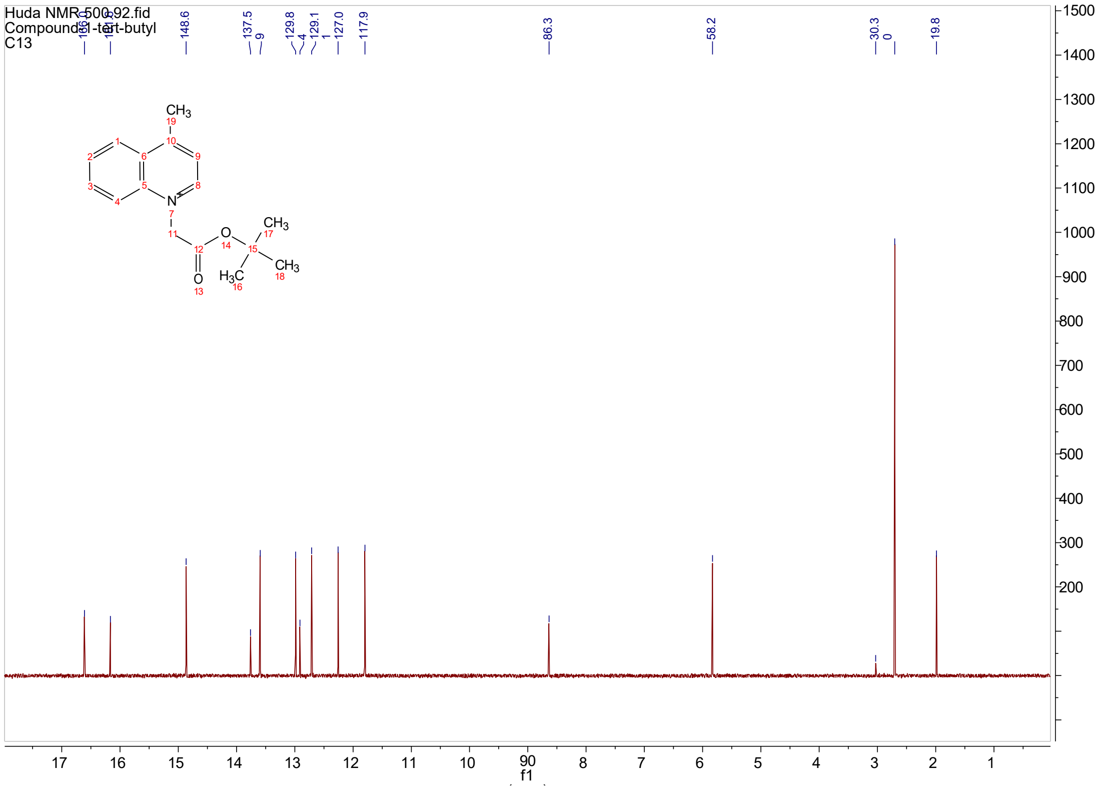

Figure S16: ^{1}H and ^{13}C NMR spectrum of compound **5**

^{1}H and ^{13}C-NMR spectra of compound 7

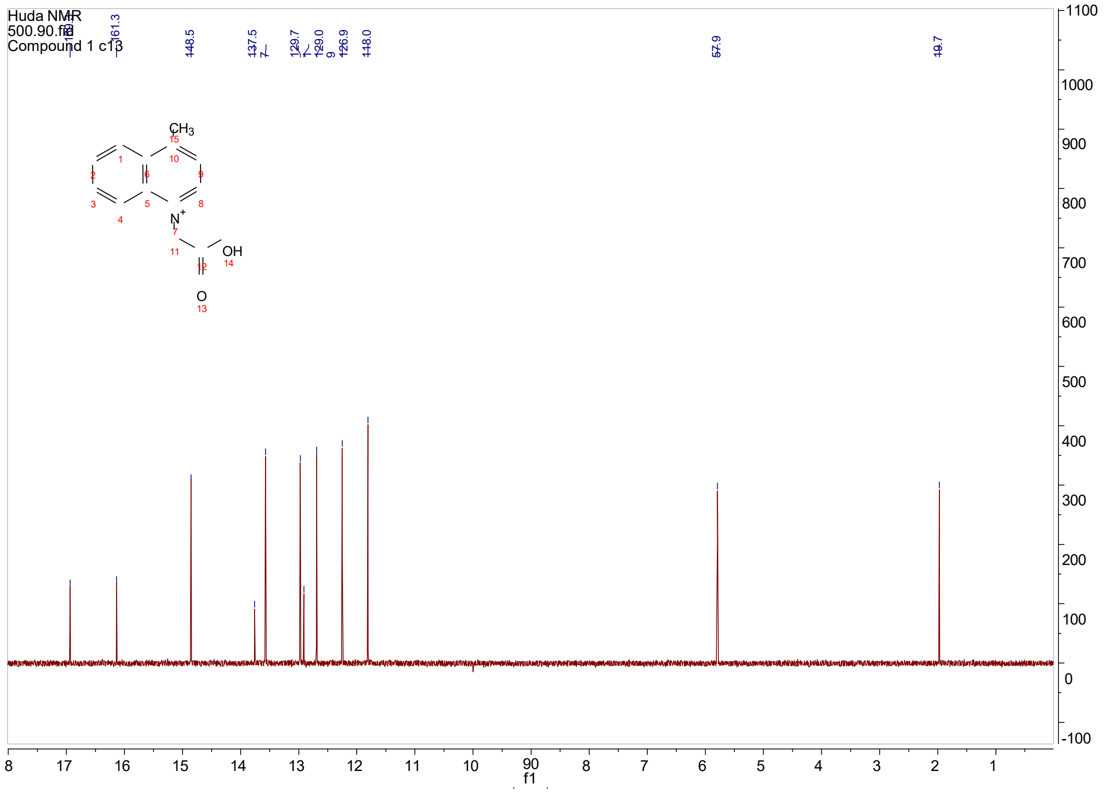

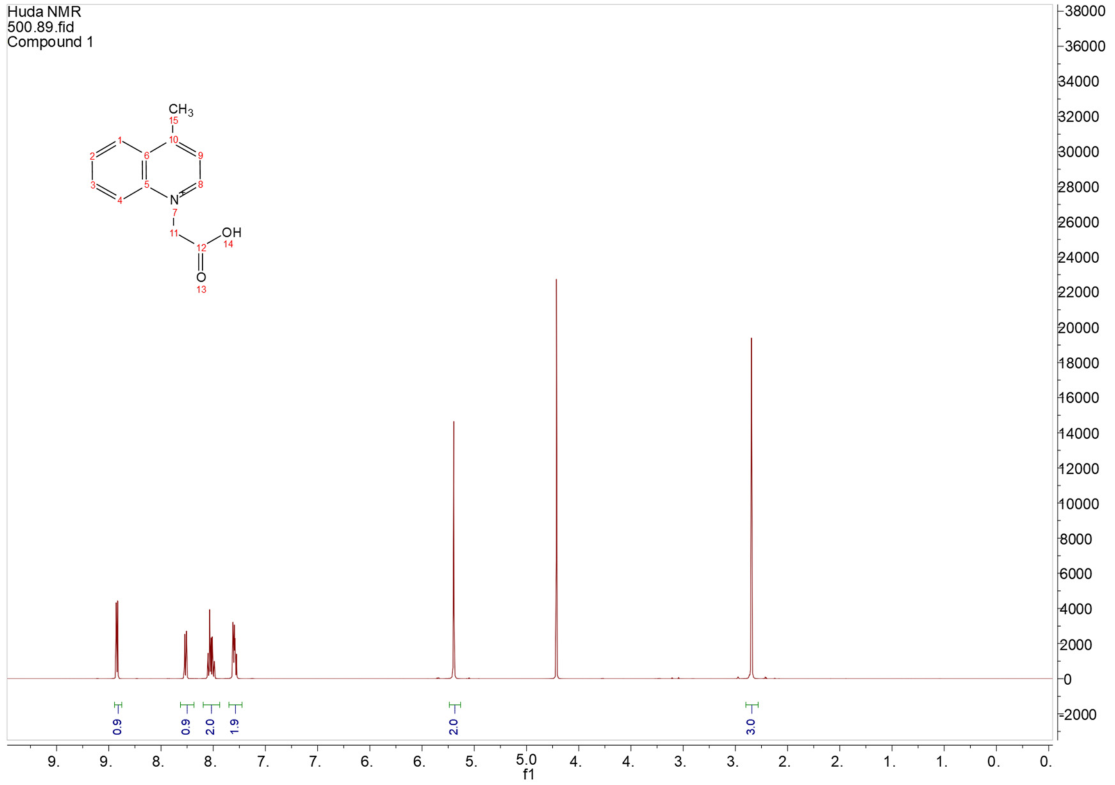

Figure S17: ^{1}H and ^{13}C NMR spectrum of compound **7**

^{1}H and ^{13}C NMR spectra of BisQ monomer

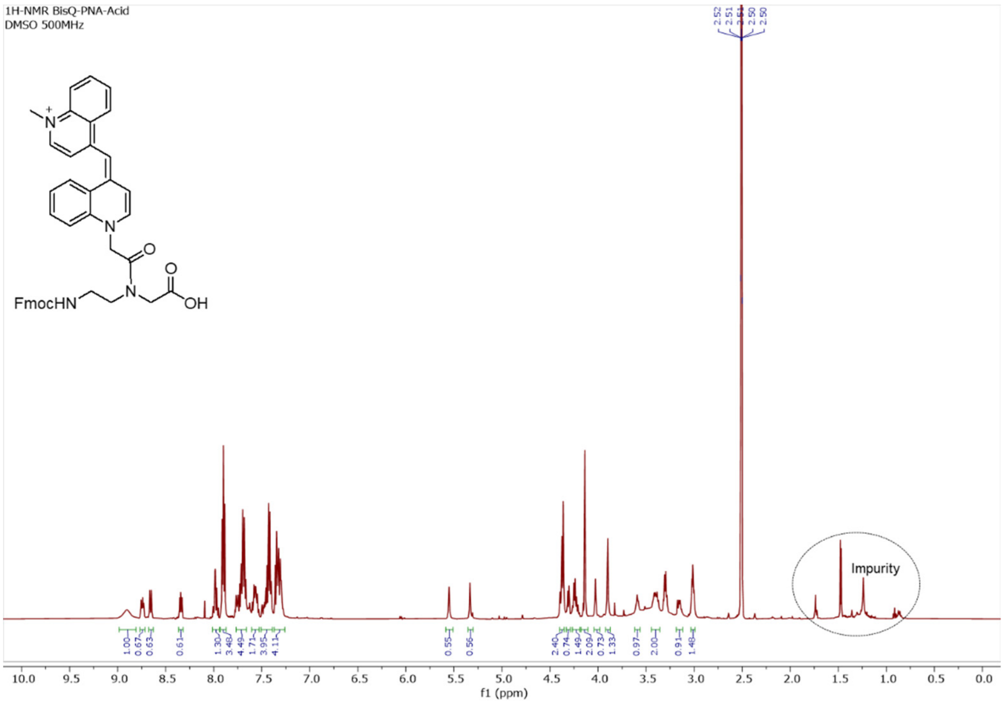

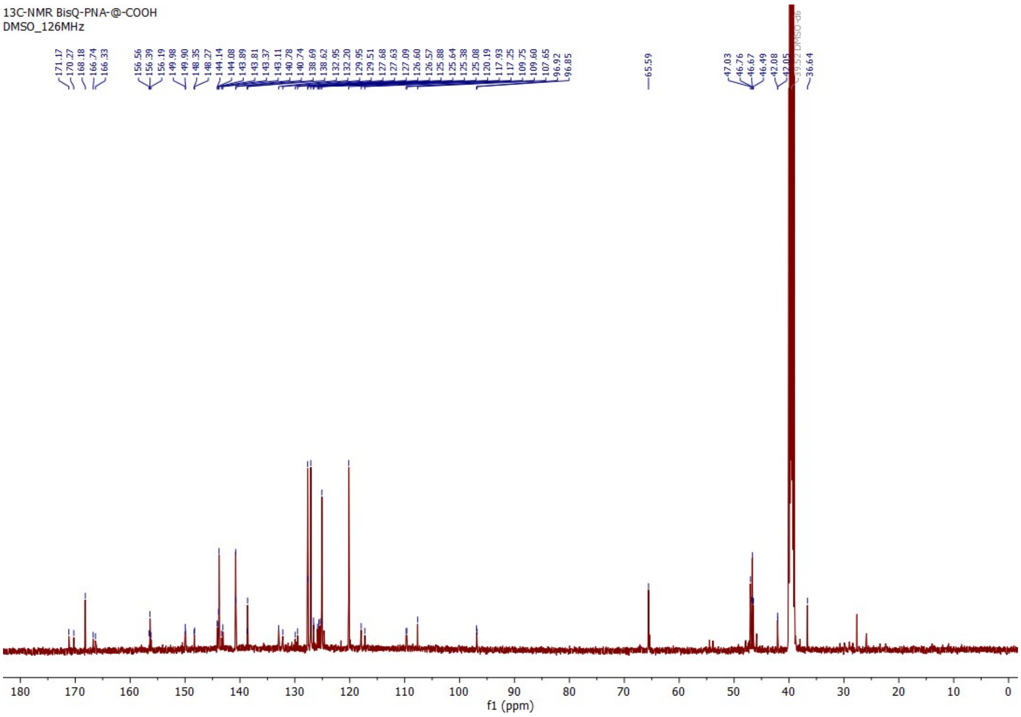

Figure S18: ^{1}H and ^{13}C NMR spectrum of **BisQ-PNA-monomer**
